# Carmi Syndrome in a Neonate: An Exacting Surgical Challenge

**DOI:** 10.7759/cureus.10522

**Published:** 2020-09-18

**Authors:** Ahmad A Al Faqeeh, Muhammad Khalid Syed, Salman Hussain, Talal Almas, Mohammed Ammar

**Affiliations:** 1 Pediatric Surgery, King Fahad Hospital, Al Bahah, SAU; 2 Internal Medicine, Royal College of Surgeons in Ireland, Dublin, IRL

**Keywords:** pyloric atresia, epidermolysis bullosa, carmi syndrome

## Abstract

Carmi syndrome is characterized by the concomitant presence of pyloric atresia and epidermolysis bullosa. Pyloric atresia routinely presents with symptoms of gastrointestinal obstruction, which include vomiting and feeding intolerance. On the other hand, epidermolysis bullosa presents with blistering skin lesions upon the slightest trauma. Due to these skin lesions, the affected patients are particularly susceptible to developing septicemia and adverse disease outcomes. We hereby delineate a case of Carmi syndrome in a neonate who was treated surgically. Postoperatively, the neonate began to deteriorate and eventually developed septicemia and passed away shortly thereafter.

## Introduction

The association of pyloric atresia (PA) and epidermolysis bullosa (EB) is extremely rare but well-documented [1]. PA has been reported to have an incidence of one in 100,000 live births and accounts for less than 1% of all gastrointestinal atresia-related disorders while the incidence of EB is one in 300,000 [2-3]. The concomitant presence of PA and EB is known as Carmi syndrome [4]. Carmi syndrome is characterized by skin fragility and blistering with minimal, or even no, trauma, along with an obstruction of the pylorus. Notably, this rare syndrome demonstrates a predilection for affecting neonates [5]. The disease carries a high mortality rate owing to its systemic manifestations such as electrolyte imbalances and septicemia. For this reason, Carmi syndrome often poses a surgical challenge [6]. We hereby delineate the case of a pre-term infant who presented with feeding intolerance and signs of skin blistering. Further diagnostic workup revealed the presence of Carmi syndrome. The patient was treated surgically but eventually deteriorated and passed away.

## Case presentation

**Figure 1:**
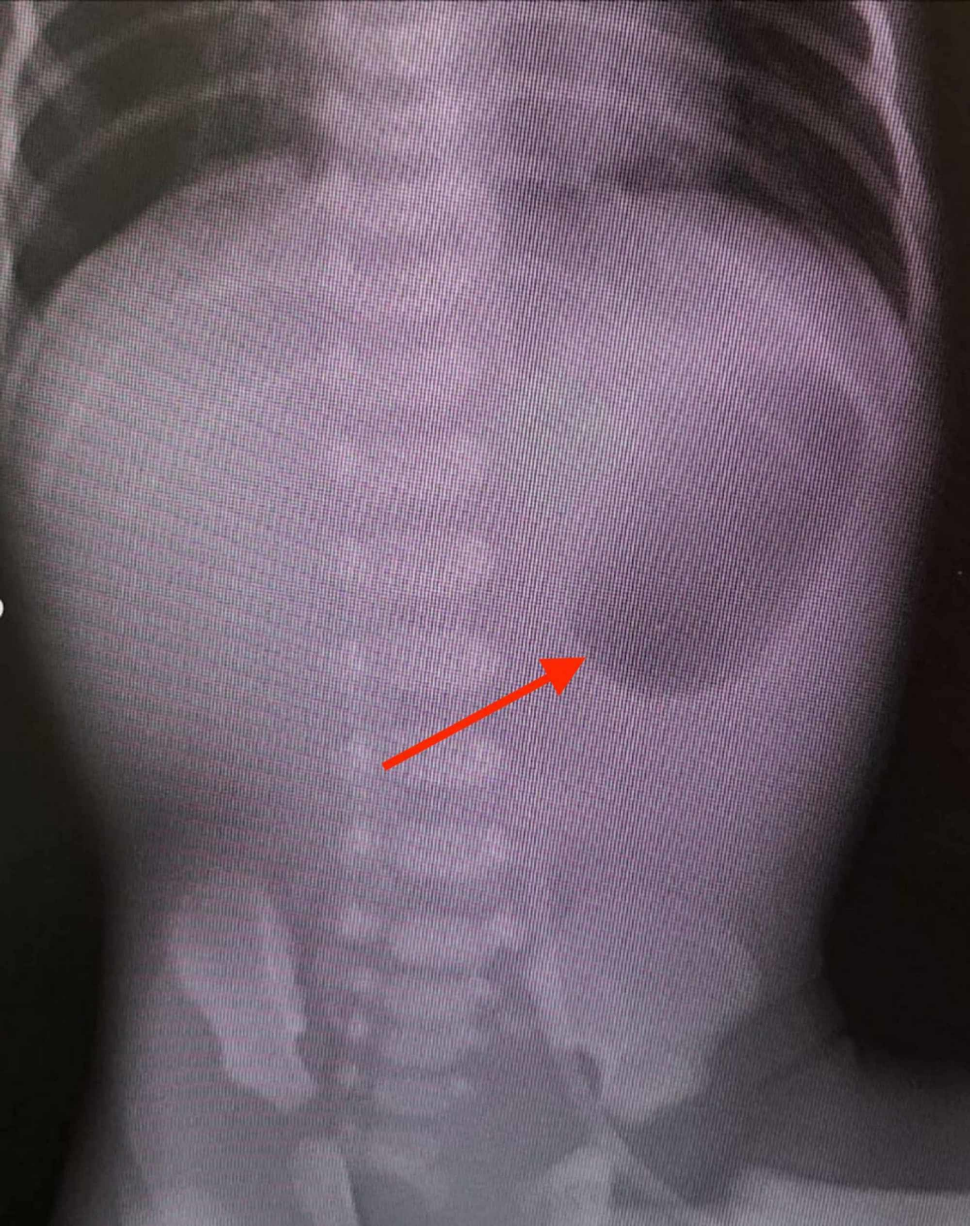
An abdominal radiograph demonstrating a single bubble sign (red arrow), which aligns with the diagnosis of pyloric atresia

We chronicle the case of a pre-term infant with a birth weight of 2 kilograms, born at the 33rd week of gestation, who presented to the neonatal intensive care unit on the second day after birth. The neonate presented with a history of feeding intolerance, which included an inability to tolerate breast and formula milk. Notably, this was on a background history of blistering skin peeling. On examination, the neonate was active, with normal vital signs. A plain abdominal radiograph revealed a single bubble sign representing a distended stomach with no distal gas presence (Figure 1).

**Figure 2:**
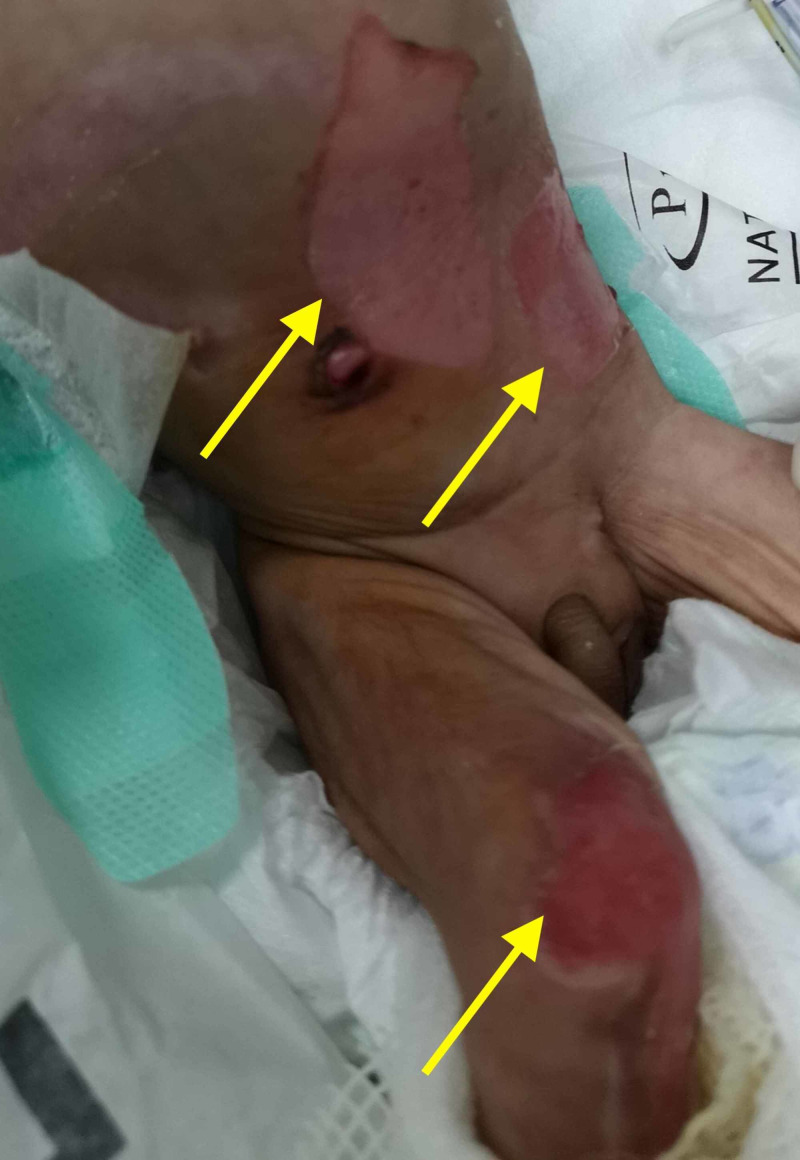
A depiction of the diffuse skin blistering (yellow arrows) as seen in the neonate

Pertinently, there was no gas present in the remainder of the gut and the chest was clear bilaterally. Physical examination further divulged the presence of diffuse skin blistering, with bullae all over the neonate's limbs, and alluded to a diagnosis of epidermolysis bullosa (Figure 2).

**Figure 3:**
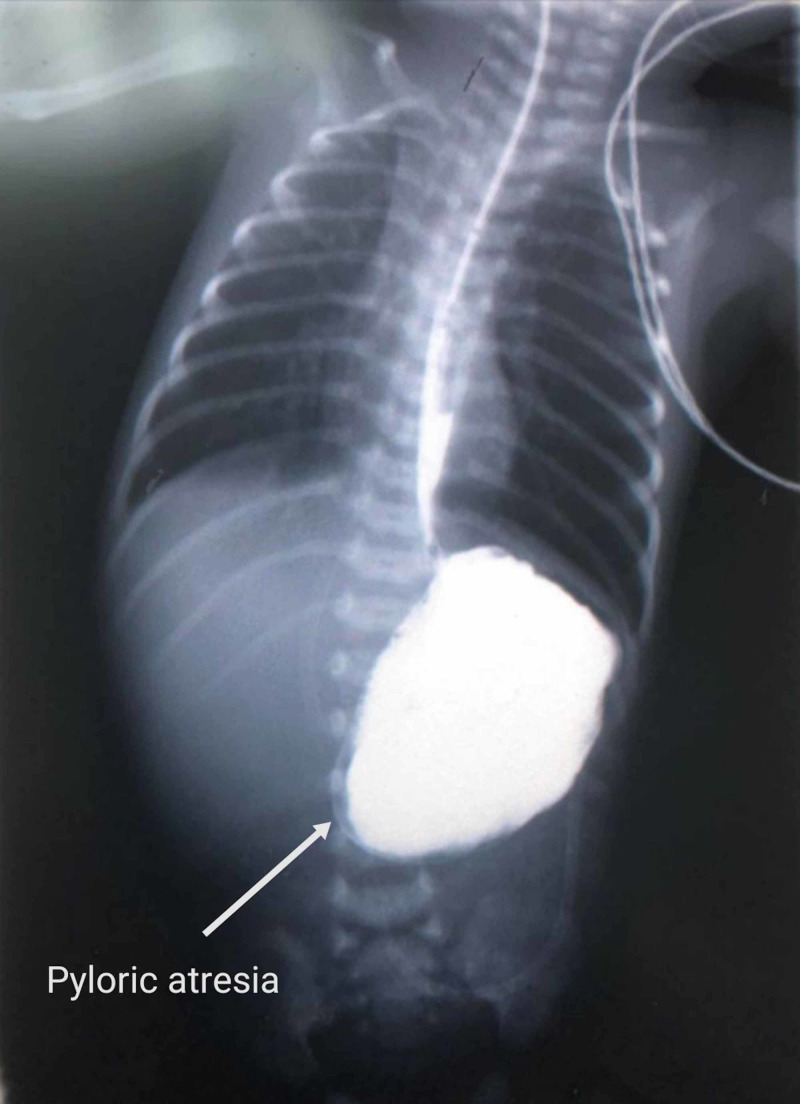
A contrast study of the upper GIT demonstrating no contrast uptake beyond the stomach and thus delineating the presence of pyloric atresia (white arrow) GIT: gastrointestinal tract

Interestingly, other associated anomalies include malformed left ear, short lower limbs, and undescended testes, which aroused the suspicion of the presence of a congenital anomaly. In addition, the mother's previous infant had passed away after suffering from the same clinical manifestations. In order to better elucidate the etiology underlying the patient's gastrointestinal symptoms, an upper gastrointestinal tract (GIT) contrast study was performed. The study revealed no contrast beyond the stomach, reaffirming the diagnosis of pyloric atresia (Figure 3).

**Figure 4:**
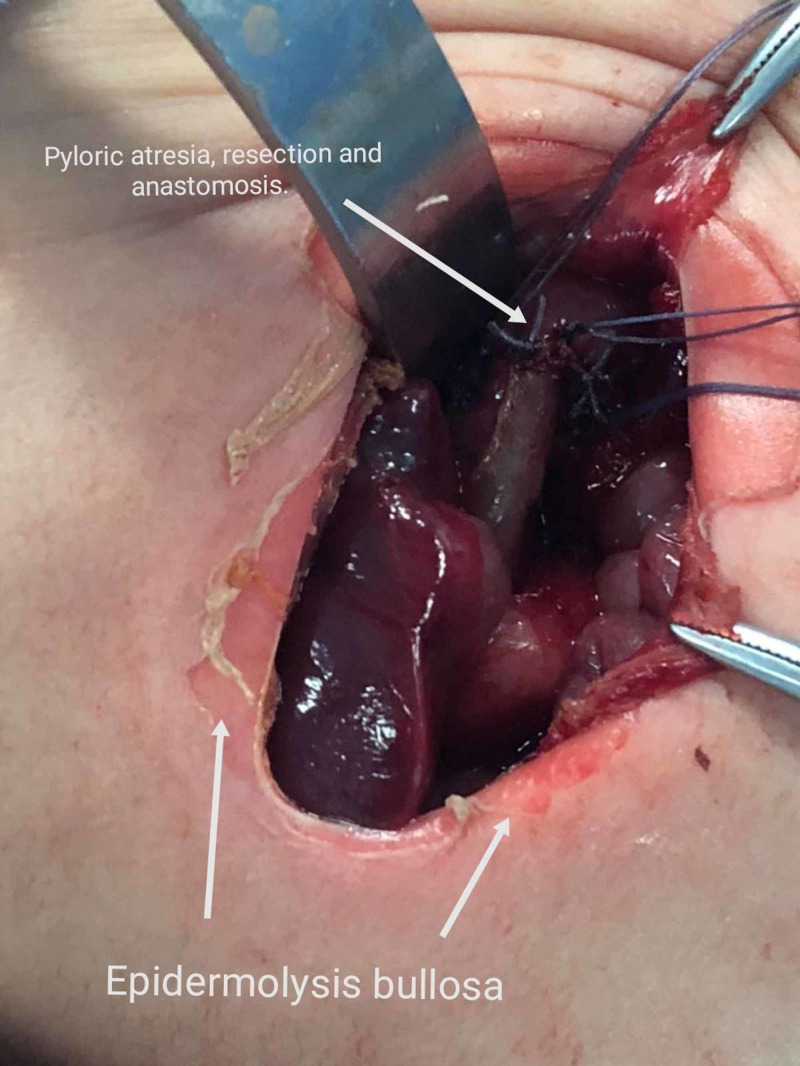
Intraoperative image showing the concomitant presence of pyloric atresia and epidermolysis bullosa An anastomosis between the distal stomach and duodenum was created.

Considering the concomitant presence of pyloric atresia and epidermolysis bullosa, a diagnosis of Carmi syndrome was made. Cognisant of the high neonatal mortality rate associated with the condition, a surgical approach was adopted. During the operation, type-2 pyloric atresia was observed, and the neonate underwent Billroth 1 operation, whereby the pylorus was excised and the distal stomach was anastomosed with the first part of the duodenum (Figure 4).

In the aftermath of the operation, the neonate recovered unremarkably, with no postoperative complications. However, the neonate's health began deteriorating on the sixth day postoperatively, with worsening skin peeling that eventually culminated in septicemia. Unfortunately, the patient succumbed on the 11th postoperative day despite the administration of all possible treatment modalities, including high-dose antibiotics.

## Discussion

PA usually presents as non-bilious vomiting soon after birth, without abdominal distension. The plain abdominal radiograph is the gold standard imaging modality for diagnosing PA and reveals a single gas bubble that is representative of the distended stomach with no distal gas. All patients with PA should be screened for multiple anomalies, including tracheoesophageal fistulas, renal and ureteral anomalies, and EB [7]. The surgical treatment of PA depends on the anatomic variety. PA displays three anatomic variations. The variation type 1 is treated by a Heineke-Mikulicz pyloroplasty, whereas the type-2 variation is usually treated through the removal of the atretic segment during a gastroduodenostomy. Similarly, the type-3 variant, which is characterized by PA with a gap between the stomach and duodenum, is treated by means of a gastroduodenostomy [2,8].

EB encompasses a group of skin disorders characterized by blistering vesicular lesions in response to minimal or no trauma [3]. While there are no definite treatment options, the management of EB focuses predominantly on the minimization of new blister formation via appropriate dressings and infection control through the use of antibiotics and antiseptics. In addition, fluid and electrolyte replenishment, along with appropriate nutritional supplementation, remains pivotal in the management of these patients [5].

The co-existence of PA and EB is referred to as Carmi syndrome and has been reported to display an autosomal recessive inheritance pattern [9]. This could explain the presence of Carmi syndrome in both of the mother's neonates in our case. Clinically, neonates present with widespread areas of blistering and the congenital localized absence of skin (aplasia cutis congenita), as well as abdominal distention and recurrent vomiting [10]. The diagnosis of Carmi syndrome should be considered in every neonate with PA regardless of the degree of skin blistering [5]. The literature has documented a very high mortality rate and poor outcomes that can mainly be attributed to the resultant sepsis. Dank et al. reported an average survival time of 70 days among 51 patients with Carmi syndrome undergoing a surgical correction, alluding to a dismal disease prognosis owing to imminent sepsis [11]. For this reason, some studies have posited that surgical correction of PA is to be withheld in order to avoid unwarranted needle-associated suffering [12]. Nevertheless, a meticulous clinical examination, along with comprehensive history-taking, can aid in yielding a prompt diagnosis of the condition.

On the other hand, Hayashi and colleagues reported four patients with survival between 17 months and 16 years [12]. In addition, Sahebpor et al. reported five patients with the syndrome, four of which were alive at the follow-up period [2]. In our case, type-2 PA was diagnosed in a patient with Carmi syndrome and was corrected surgically through a Billroth 1 procedure. Noticeable deterioration commenced on the sixth day postoperatively, with worsening peeling of the skin. Eventually, the patient succumbed to the aggressive disease and passed away on the 11th postoperative day.

## Conclusions

Carmi syndrome refers to the concomitant presence of pyloric atresia and epidermolysis bullosa and is noted to portend an exceedingly dismal prognosis in the neonatal population. Due to the diffuse and extensive skin blistering noted, septicemia routinely ensues. Emergent surgical intervention, without any preoperative delays, therefore remains focal in yielding favorable disease outcomes.
